# Gender Differences in Exercise Dependence and Eating Disorders in Young Adults: A Path Analysis of a Conceptual Model

**DOI:** 10.3390/nu6114895

**Published:** 2014-11-05

**Authors:** Shelli Meulemans, Peter Pribis, Tevni Grajales, Gretchen Krivak

**Affiliations:** 1 School of Health Professions, Department of Public Health & Wellness, Andrews University, 8475 University Boulevard—Marsh Hall, Berrien Springs, MI 49104-0210, USA; E-Mails: smeulemans@lakelandregional.org (S.M.); krivak@andrews.edu (G.K.); 2 College of Education, Department of Individual, Family & Community Education, University of New Mexico, Hokona Hall 156 MSC05 3040, Albuquerque, NM 87131-0001, USA; 3 School of Education, Department of Educational & Counseling Psychology, Andrews University, 4195 Administration Drive—Bell Hall 159, Berrien Springs, MI 49104-0104, USA; E-Mail: tevni@andrews.edu

**Keywords:** gender, exercise dependence, eating disorder, mediator

## Abstract

The purpose of our study was to study the prevalence of exercise dependence (EXD) among college students and to investigate the role of EXD and gender on exercise behavior and eating disorders. Excessive exercise can become an addiction known as exercise dependence. In our population of 517 college students, 3.3% were at risk for EXD and 8% were at risk for an eating disorder. We used Path analysis the simplest case of Structural Equation Modeling (SEM) to investigate the role of EXD and exercise behavior on eating disorders. We observed a small direct effect from gender to eating disorders. In females we observed significant direct effect between exercise behavior (*r* = −0.17, *p* = 0.009) and EXD (*r* = 0.34, *p* < 0.001) on eating pathology. We also observed an indirect effect of exercise behavior on eating pathology (*r* = 0.16) through EXD (*r* = 0.48, *r*^2^ = 0.23, *p* < 0.001). In females the total variance of eating pathology explained by the SEM model was 9%. In males we observed a direct effect between EXD (*r* = 0.23, *p* < 0.001) on eating pathology. We also observed indirect effect of exercise behavior on eating pathology (*r* = 0.11) through EXD (*r* = 0.49, *r*^2^ = 0.24, *p* < 0.001). In males the total variance of eating pathology explained by the SEM model was 5%.

## 1. Introduction

Excessive exercise can become an addiction known as exercise dependence (EXD). EXD occurs when the person is preoccupied with exercise, has withdrawal symptoms upon cessation, continues to exercise when socially or medically contraindicated, and when exercise interferes with relationships and work [1,2,3,4,5,6,7,8].

Studies have been conducted on the prevalence of EXD and eating disorders. However, there is debate on the methodologies, diagnosis cut off points, and the relationship between the two [3,9,10,11,12,13]. Although eating disorders are recognized by the *American Psychiatric Association* in the *Diagnostic and Statistical Manual of Mental Health Disorders* (DSM-V), EXD as a disorder on its own, has not been officially categorized [1]. There are two proposed variants of EXD: primary and secondary. Primary exercise dependency is defined as when individual meets the criteria for EXD and continually exercises solely for the psychological gratification resulting from the exercise behavior [14]. Secondary EXD is defined as occurring when an exercise dependent individual uses excessive exercise to accomplish some other end, such as weight loss; secondary EXD occurs in conjunction with other pathologies like several variants of eating disorders, body or muscle dysmorphia [2,15]. Research indicates that people who are at high risk for developing EXD are high-performing athletes, young women, former athletes, high achievers, and those with body image issues or addictive personalities [16].

Eating disorders are psychological disorders that involve a persistent disturbance in eating patterns or other behaviors intended to control weight, body size or shape. Eating disorders affect physical and nutritional health, psychological functioning and if untreated, they can be fatal. According to mental illness classification system, there are three categories of eating disorders: anorexia nervosa, bulimia nervosa, and Eating Disorders Not Otherwise Specified (EDNOS), which include binge-eating disorder [17]. Genetic, psychological, and sociocultural factors contribute to the development of eating disorders [18,19]. Gender is a very important factor in eating pathology; women are more likely than men to develop eating disorders. In the United States, eating disorders effect about 3% of girls and women and 1% of boys and men [20,21]. Similar to EXD, the greatest frequency of eating disorders is found in groups concerned about maintaining low body weight, such as dancers, gymnasts, figure skaters, wrestlers and models [22]. Significant ethnic differences have been reported for bulimia nervosa, with Hispanic adolescent reporting the highest prevalence; ethnic minorities reporting more binge-eating disorder, and Caucasian adolescent tending more toward anorexia nervosa [23]. Adherence to a vegetarian diet has been hypothesized to be a factor in the onset and maintenance of disordered eating behavior; however, evidence to support this assumption has been mixed. A recent study by Timko *et al.* suggests that true vegetarians and vegans appear to be the healthiest in regards to weight and eating and semi-vegetarianism is the most likely to be related to disordered eating [24].

In summary, EXD is a problematic behavior, which can affect individuals with eating disorders. A review of previous research indicates that EXD could possibly mediate the relationship between exercise behavior and eating pathology [9,10], but some of the studies were done only on females. It is thus not clear, what is the mechanism between exercise behavior, EXD and eating disorders.

Therefore, using a nonclinical adolescent ethnically diverse sample, with a wide range of dietary preferences, the goal of this cross-sectional observational study was to (i) report the prevalence of EXD among college students (ii) to investigate the role of EXD and exercise behavior on eating disorders and (iii) to observe the role of gender on eating disorders.

## 2. Experimental Section

### 2.1. Participants

With the approval of the University’s Institutional Review Board, students in various undergraduate and graduate classes on the campus of Andrews University were asked to complete a self-reported, anonymous, paper based questionnaire. The questionnaire was composed of four sections: 14 demographic and exercise behavior questions, a 31-item Food Frequency Questionnaire (FFQ), the Exercise Dependency Scale Test (EDS-21), and the Eating Attitudes Test (EAT-26). There were 567 participants who completed the survey; 47 subjects were disqualified because they were not between ages 18 to 25, three subjects were removed because of missing data leaving a study population of 517 (mean age 19.7 ± 1.7; 44% males, 56% females), representing a wide range of ethnic backgrounds (37% Caucasian, 22% African American, 12% Asian, 12% Hispanic and 17% mixed ethnicity), class standing (50% Freshman, 21% Sophomore, 15% Junior, 12% Senior, and 2% Graduate students) and dietary preferences (63% omnivore, 37% vegetarian). Andrews University is a Seventh-day Adventist (SDA) institution of higher learning. SDAs represent a unique population because this religious group actively endorses a healthy lifestyle.

### 2.2. Measures

The EDS-21 is based on the DSM-IV criteria for substance dependence. It provides the following information: mean overall score of exercise dependence symptoms, and the differentiation between: (a) at-risk for exercise dependence; (b) nondependent-symptomatic; and (c) nondependent-asymptomatic. The items on the scale are based on the following criteria: tolerance, withdrawal, intention effect, lack of control, time, reductions in other activities, and continuance. The scale involves 21 items on a Likert scale ranging from 1 (never) to 6 (always). The scale has acceptable test-retest and internal consistency reliability, content and concurrent validity [25,26].

The Eating Attitudes Test (EAT-26) consists of 26 questions on the 6-point Likert scale ranging from 1 (always) to 6 (never). It is useful and valid for determining the prevalence of an eating disorder but not diagnosing the specific disorder. A score of greater than or equal to 20 is considered “at-risk” for an eating disorder. The EAT-26 has been validated and can be regarded as a reliable and valid instrument for screening for eating disorders [9,27].

Exercise behavior was ascertained using four questions (on average how many hours a week do you spend in aerobic exercise; on average how many hours a week do you spend in strength training exercise; on average how many hours a week do you spend in flexibility exercise; what type of aerobic activities do you do most frequently) about the usual exercise habits of the participants. The questionnaire measured the frequency that an individual engages in aerobic, strength and flexibility exercises and the type of aerobic activities done most frequently. Each score was then converted into metabolic equivalents (METS) (aerobic exercise × 7, strength training × 6, and flexibility exercise × 2.5) and summed up to provide the estimated energy expenditure in METS/week [28].

The FFQ was used to accurately ascertain the vegetarian status of the participants.

### 2.3. Statistical Analysis

The data was analyzed using SPSS (version 18.0; SPSS Inc., Chicago, IL, USA) for descriptive statistics and AMOS 7.0 statistical software for SEM. Structural Equation Modeling (SEM) is a statistical procedure that enables researchers to evaluate specific relationships hypothesized, and Path analysis is the simplest case of SEM where we do not include latent variables [29]. Raw data was initially described and explored in order to identify missing and outlier cases and to test the normal distribution assumptions. Two different models were tested. The initial model examined the direct effects of exercise behavior, EXD and gender on eating pathology. An alternative model examined additional indirect effects of exercise behavior on eating pathology. We also performed gender specific model testing. Model comparisons were based on chi-square differences. Model modifications to determine the best-fit model were based on theoretical, as well as statistical, judgments (Comparative Fit Index (CFI), Tucker-Lewis Index (TLI), Root Mean Square Error of Approximation (RMSEA)). *p* Values less than or equal to 0.05 were considered statistically significant.

## 3. Results

In our population, 3.3% of students were characterized as at-risk for EXD, 51.5% as nondependent-symptomatic, and 45.2% as nondependent-asymptomatic. The prevalence of at-risk (EAT-26 score > 20) for an eating disorder was 8%. The EAT-26 test includes four behavioral questions regarding possible eating disorder symptoms; 10% of participants admitted binging, 2.9% admitted vomiting, 3.7% admitted the use of laxatives, diet pills or diuretics and 2.1% admitted being treated for an eating disorder in the last six months. Table 1 shows selected characteristics of the participants by their EXD status. Participants at-risk for EXD were mostly male, Hispanic or Caucasian, spent more time and energy weekly exercising, and also scored higher on EAT-26 and EDS-21 scores. The most popular sport among those at-risk for EXD was running and walking.

### 3.1. Preliminary Analysis

In our preliminary analysis we followed the general analytic consideration suggested by Baron and Kenny [30] to replicate the potential mediation of EXD in a three-variable (EAT-26, EXD, METS) model. However, in our study in both genders combined, and in females, the fundamental assumption that all variables must be significantly correlated was not satisfied; there was no significant correlation between METS and EAT-26. However, we were able to show a mediator effect of EXD on the relationship between METS and eating pathology for males, but only using the raw data. One of the underlying assumptions of regression is that variables should be approximately normally distributed [31]. After correcting the normality problem of METS with log transformation, the mediating effect of EXD in males disappeared. In our population, EXD did not mediate the relationship between exercise behavior and eating pathology either in males nor females.

**Table 1:** Characteristics of participants by their EXD status.

Variable Categories	At-Risk	Nondependent Symptomatic	Nondependent Asymptomatic
	(*n* = 17)	(*n* = 268)	(*n* = 235)
Males (*n*, %)	10 (59)	127 (48)	89 (38)
Caucasian (*n*, %)	4 (24)	111 (42)	78 (34)
African American (*n*, %)	3 (18)	54 (21)	57 (25)
Hispanic (*n*, %)	6 (35)	30 (12)	25 (11)
Asian (*n*, %)	1 (6)	29 (11)	29 (12)
Mixed ethnicity (*n*, %)	3 (17)	37 (14)	43 (18)
Aerobic exercise: hours/week (mean ± SD) *	6.1 ± 5.1 ^†^	3.9 ± 3.2 ^†,‡^	2.3 ± 2.5 ^†,‡^
Strength training: hours/week (mean ± SD) *	5.9 ± 4.4 ^†^	2.8 ± 2.6 ^†,‡^	1.0 ± 1.5 ^†,‡^
Flexibility exercise: hours/week (mean ± SD) *	2.2 ± 2.6 ^†^	1.6 ± 2.8 ^‡^	0.9 ± 1.5 ^†,‡^
METS/week (mean ± SD) *	84.1 ± 51.6 ^†^	49.0 ± 35.9 ^†,‡^	25.2 ± 24.0 ^†,‡^
EAT-score (mean ± SD) *	14.4 ± 10.3 ^†^	8.6 ± 7.6 ^†^	6.9 ± 6.1 ^†^
EDS-21 score (mean ± SD) *	94.8 ± 11.6 ^†^	57.6 ± 11.4 ^†,‡^	32.0 ± 7.6 ^†,‡^

Signifies significant trend across variable categories at *p* < 0.05; ANOVA; Post-hoc comparison using LSD test; ^†,‡^ indicates significant differences between groups; EAT-score of 20 or higher indicates at-risk for eating disorder; METS mean metabolic equivalents; EAT-26 means Eating Attitudes Test; EDS means Exercise Dependence Scale; SD means standard deviation.

### 3.2. Exploratory Analysis

Table 2 shows descriptive characteristics for exercise behavior, EXD and EAT-26 for males and females. Exploratory analysis of the data showed that the exercise behavior variable is not normally distributed. We have done a log transformation for exercise behavior in order to achieve normality following Hair *et al.* [32].

### 3.3. Model Specification

**Figure 1:**
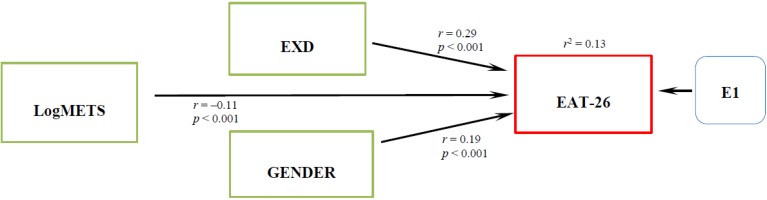
Initial Path analysis of the relationship between LogMETS, EXD, gender and EAT-26.

Initial structural model examining the direct effect of the METS, EXD and gender on eating pathology was tested by SEM techniques as it is shown in Figure 1. This first structural model was not a good fit (χ^2^ (Df = 3) 155.238, *p* = 0.000, CFI = 0.249, TLI = 0.502, RMSEA = 0.314) however significant effects were observed from the independent variables to the dependent.

### 3.4. Model Modification

**Figure 2:**
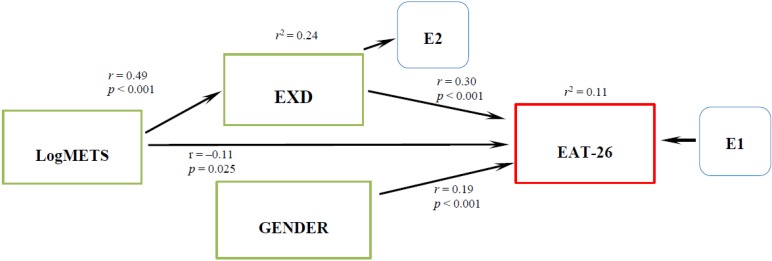
Final Path analysis of the relationship between LogMETS, EXD, gender and EAT-26.

Following an exploratory approach, an alternative model was developed using the same variables with an additional direct effect of METS on EXD, suggesting an indirect effect of METS on eating pathology. In this case, the model improved and was a good fit, (χ^2^ (Df = 2) 12.499, *p* = 0.002, CFI = 0.948, TLI = 0.845, RMSEA = 0.101) showing a significant decrease in the chi-square value. Figure 2 shows the model with coefficients and the direct and indirect effects represented by one-head arrows. Significant direct effect was observed between gender (*r* = 0.19, *p* < 0.001), METS (*r* = −0.11, *p* = 0.025) and EXD (*r* = 0.30, *p* < 0.001) on eating pathology. Finally there is an indirect effect of METS on eating pathology (*r* = 0.15) through EXD (*r* = 0.49, *r*^2^ = 0.24, *p* < 0.001). The total variance of eating pathology explained by this model is 11%.

**Table 2:** Descriptive characteristics for EAT-26, EXD, METS and LogMETS.

Descriptive Characteristics	Mean ± SD	SE	Skewness	Kurtosis
**Both Sexes**				
EAT-26	8.1 ± 7.3	0.3	1.9	4.7
EXD	47.3 ± 18.2	0.8	0.7	0.4
METS	39.5 ± 34.7	1.5	2.0	5.8
Log METS	1.4 ± 0.5	0.02	−1.3	1.6
**Males**				
EAT-26	6.6 ± 6.3	0.4	2.6	9.6
EXD	50.0 ± 18.9	1.2	0.5	−0.06
METS	45.5 ± 36.9	2.5	1.6	3.8
Log METS	1.4 ± 0.4	0.03	−1.4	2.4
**Females**				
EAT-26	9.1 ± 7.8	0.4	1.6	3.0
EXD	45.3 ± 17.3	1.0	0.9	1.1
METS	35.2 ± 32.2	1.9	2.5	8.8
Log METS	1.3 ± 0.5	0.03	−1.3	1.3

METS mean metabolic equivalents; EAT-26 means Eating Attitudes Test; EXD means Exercise Dependence; SE means Standard Error of the Mean.

### 3.5. Gender Specific Analysis

**Figure 3:**
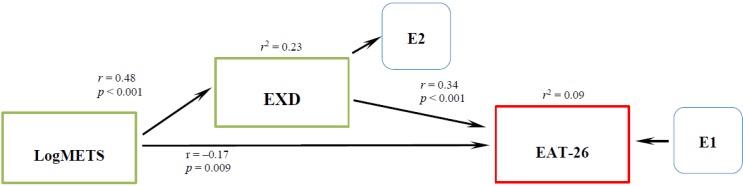
Final Path analysis of the relationship between LogMETS, EXD and EAT-26 for females.

Figure 3 shows the default (saturated) model for females was a good fit (χ^2^ (Df = 0) 0.000, CFI = 1.000) with coefficients and the direct and indirect effects. Significant direct effect was observed between METS (*r* = −0.17, *p* = 0.009) and EXD (*r* = 0.34, *p* < 0.001) on eating pathology. Finally there is an indirect effect of METS on eating pathology (*r* = 0.16) through EXD (*r* = 0.48, *r*^2^ = 0.23, *p* < 0.001). The total variance of eating pathology explained by this model is 9%.

**Figure 4:**
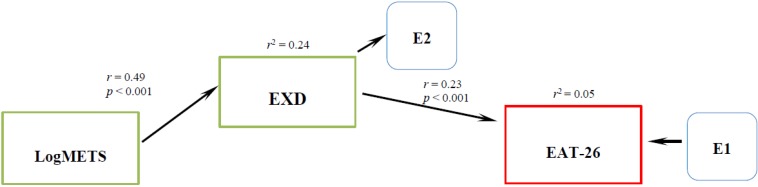
Final Path analysis of the relationship between LogMETS, EXD and EAT-26 for males.

Figure 4 shows the default model for males was a good fit (χ^2^ (Df = 1) 0.033, *p* = 0.856) with coefficients and the direct and indirect effects. Significant direct effect was observed between EXD (*r* = 0.23, *p* < 0.001) on eating pathology. There is an indirect effect of METS on eating pathology (*r* = 0.11) through EXD (*r* = 0.49, *r*^2^ = 0.24, *p* < 0.001). The total variance of eating pathology explained by this model is 5%.

## 4. Discussion

The first goal of our study was to study the prevalence of EXD among college students, to investigate the relationship between METS, EXD, gender and eating disorders, and to replicate previous research [9]. In our population, 3.3% of students could be characterized as at-risk for EXD, confirming results reported in other studies with similar rates (1%–4%) [13,16]. In our study, vegetarian status was not related to EXD or eating pathology in either males or females. The majority of those at-risk for EXD were runners or walkers, which is not surprising considering the addictive potential of running and its history as a positive addiction [6]. The majority of those at-risk for EXD were Hispanics, followed by Caucasians. Perhaps this can be attributed to the self-image differences between ethnic groups, such as Hispanics and Caucasians compared to African American and Asians. In our population, the prevalence of at-risk for an eating disorder was 8%, and the prevalence of confirmed eating disorders was 2.1% indicating similar rates reported by others [33]. Only 23% of the individuals at-risk for EXD were also considered at-risk for an eating disorder.

The second goal of our study was to investigate the role of EXD and exercise behavior on eating disorders. A mediator variable is one that explains the relationship between two other variables [30]. The study done by Cook & Hausenblas [10] targeted only females and suggested that EXD operates as mediator between METS and eating pathology. Our study was more inclusive with college age individuals of both genders. But, in our study, the fundamental assumption that all variables must be significantly correlated was not satisfied. In our population, EXD did not mediate the relationship between exercise behavior and eating pathology in analysis with both genders combined and also in the gender specific analysis.

In the next step, we proceeded to use the SEM method to test the effects of METS, EXD, and gender on eating pathology as an initial model (Figure 1). This model represents a multiple linear regression equivalent that explains the variability of eating pathology by the direct effects of METS, EXD and gender as predictors. However, this model did not fit indicating that the eating pathology model needs some improvement. In the next step we found that an additional indirect effect of METS on eating pathology through EXD satisfied the model fit criteria (Figure 2). We discovered that METS could impact eating pathology in two different ways. The direct effect of METS on eating pathology is a negative effect, suggesting that people with high scores on exercise behavior have lower scores in eating pathology. Healthy exercise behavior apparently does not promote eating disorders. However, through the indirect effect, people with high scores in exercise behavior and high scores in ED also have high scores in eating pathology. Unhealthy exercise behavior as observed by EXD nevertheless, seems to explain eating pathology. This finding is observed controlling with the gender variable, showing the significance of gender in eating pathology. This agrees with previous research showing significant differences in eating disorders by gender [34]. We proceeded to perform a gender specific analysis; for females we observed a direct and an indirect effect of exercise behavior on eating pathology, (Figure 3) similar to the one described by Cook and Hausenblas [10], however in males the METS only has an indirect effect on eating pathology (Figure 4).

The third goal of our study was to investigate the role of gender on eating disorders. We did observe a small direct effect (*r* = 0.19, *p* < 0.001) from gender to eating disorders (Figure 2). In our study we could not confirm neither moderating nor mediating effect of exercise behavior on EXD and eating pathology as reported by others [10,35,36]. Nevertheless, the different pathways observed in the SEM gender specific models (Figure 3 and Figure 4) between exercise behavior, EXD and eating pathology perhaps could indicate different treatment approaches for different genders.

Several potential limitations to this study should be considered. This is a population-based cross-sectional study, which included both genders. Our study population was non-clinical, self-reported, with low incidence of EXD. The study was conducted on a campus of an American private university, which may limit the generalizability of the results. Although SEM is a sophisticated analytic tool for testing theoretical models in behavioral or social science, the analyses are correlational which makes it difficult to establish causality. Because the isolation of variables in the models is impossible, all models must be looked at only as an estimation of reality [37]. We used different instruments for eating pathology and exercise behavior than other studies, which could contribute to variability in results. Our exercise behavior questionnaire was self-reported and not validated; however it was based on principle following the Leisure-time Exercise Questionnaire [38].

More research is needed to replicate our results using both genders and different (clinical and nonclinical) populations to clarify the relationship of exercise behavior and eating disorders.

## 5. Conclusions

In conclusion in our population, 3.3% of students were characterized as at-risk for EXD, 51.5% as nondependent-symptomatic, and 45.2% as nondependent-asymptomatic. The prevalence of at-risk (EAT-26 score > 20) for an eating disorder was 8%; 10% of participants admitted binging, 2.9% admitted vomiting, 3.7% admitted the use of laxatives, diet pills or diuretics and 2.1% admitted being treated for an eating disorder in the last six months.

In our population significant direct effect was observed between gender, METS and EXD on eating pathology and an indirect effect of METS on eating pathology through EXD.

In our population of females, we observed a significant direct effect between exercise behavior and EXD on eating pathology and an indirect effect of exercise behavior on eating pathology through EXD. In males, we observed a significant direct effect between EXD on eating pathology and an indirect effect of exercise behavior on eating pathology through EXD. Given the limitations of the study, the different pathways observed in the SEM gender specific models between exercise behavior, EXD and eating pathology perhaps could indicate different treatment approaches for different genders.
